# Morphological and Photosynthetic Parameters of Green and Red Kale Microgreens Cultivated under Different Light Spectra

**DOI:** 10.3390/plants12223800

**Published:** 2023-11-08

**Authors:** Barbara Frąszczak, Monika Kula-Maximenko, Anna Podsędek, Dorota Sosnowska, Kingsley Chinazor Unegbu, Tomasz Spiżewski

**Affiliations:** 1 Department of Vegetable Crops, Poznań University of Life Sciences, Dąbrowskiego 159, 60-594 Poznań, Poland; k.unegbu@gmail.com (K.C.U.); tomasz.spizewski@up.poznan.pl (T.S.); 2 The Franciszek Górski Institute of Plant Physiology, Polish Academy of Sciences, ul. Niezapominajek 21, 30-239 Kraków, Poland; m.kula@ifr-pan.edu.pl; 3 Institute of Molecular and Industrial Biotechnology, Faculty of Biotechnology and Food Sciences, Lodz University of Technology, Stefanowskiego 2/22, 90-537 Łódź, Poland; anna.podsedek@p.lodz.pl (A.P.); dorota.sosnowska@p.lodz.pl (D.S.)

**Keywords:** *Brassica oleracea* var. *acephala* L., blue light, red light, LEDs

## Abstract

Microgreens are plants eaten at a very early stage of development, having a very high nutritional value. Among a large group of species, those from the Brassicaceae family, including kale, are very popularly grown as microgreens. Typically, microgreens are grown under controlled conditions under light-emitting diodes (LEDs). However, the effect of light on the quality of grown microgreens varies. The present study aimed to determine the effect of artificial white light with varying proportions of red (R) and blue (B) light on the morphological and photosynthetic parameters of kale microgreens with green and red leaves. The R:B ratios were for white light (W) 0.63, for red-enhanced white light (W + R) 0.75, and for white and blue light (W + B) 0.38 at 230 µmol m^−2^ s^−1^ PPFD. The addition of both blue and red light had a positive effect on the content of active compounds in the plants, including flavonoids and carotenoids. Red light had a stronger effect on the seedling area and the dry mass and relative chlorophyll content of red-leaved kale microgreens. Blue light, in turn, had a stronger effect on green kale, including dry mass. The W + B light combination negatively affected the chlorophyll content of both cultivars although the leaves were significantly thicker compared to cultivation under W + R light. In general, the cultivar with red leaves had less sensitivity to the photosynthetic apparatus to the spectrum used. The changes in PSII were much smaller in red kale compared to green kale. Too much red light caused a deterioration in the PSII vitality index in green kale. Red and green kale require an individual spectrum with different proportions of blue and red light at different growth stages to achieve plants with a large leaf area and high nutritional value.

## 1. Introduction

The popularity of microgreens as a new type of superfood is continuously increasing [1]. There are several factors contributing to this. First, microgreens are easy to grow and both large-scale and amateur cultivation is possible [2]. Second, it is estimated that around 100 species of agricultural, vegetable, and herbaceous plants are grown as microgreens [3], which offer a wide variety and choice. The different species also offer a range of taste and colour qualities. Another factor contributing to the growing popularity of microgreens is their high nutritional value. Numerous studies show that the content of active compounds, vitamins, and mineral elements is higher in the herb of microgreens compared to mature plant forms [4,5,6]. The most challenging issues in cultivation are harvesting and making the postharvest shelf life of the plants as long as possible [7]. This is why plants are often sold in the boxes in which they were grown.

The Brassicaceae family includes a wide range of species commonly used in micro-scale vegetable production [8]. The colour, aroma, flavour, and health-promoting properties of brassica vegetables depend on the presence of specific secondary plant metabolites and their concentration in plant tissues [9]. Brassica vegetable microgreens are rich sources of bioactive compounds such as glucosinolates (GSLs), anthocyanins, polyphenols, ascorbic acid, carotenoids, and tocopherols [10,11]. Therefore, plants in the Brassicaceae family are widely recognised as rich sources of health-promoting phytochemicals [12], and include a range of vegetables consumed worldwide, including kale (*Brassica oleracea* L. var. *acephala* DC), which is rich in glucosinolates, minerals, carotenoids, and vitamins [11,12,13,14].

It is common for microgreens to be grown in so-called closed systems under completely controlled conditions without sunlight, only using artificial light [15]. Light can significantly modify the morphological characteristics as well as the content of active compounds in the herb of microgreens, which is why the selection of an appropriate light spectrum is one of the key factors in this type of cultivation [16]. Since chlorophyll pigments absorb mainly red (663 nm and 642 nm) and blue (430 nm and 453 nm) light, these are the main light wavelengths affecting plant growth. Red light supplemented with blue light increases photosynthetic capacity and biomass accumulation in plant seedlings [17,18]. The combination of blue and red LEDs is widely used in the production of horticultural plants, including microgreens [3,19,20]. In general, blue as well as red light regulates many aspects of morphogenesis, including shoot elongation, cell differentiation, the modulation of shoot growth, and flowering initiation [21]. Previous studies have indicated that the morphological parameters of microgreens vary according to the proportion of blue/red light provided by LEDs. For example, among the 28 species of microgreens analysed, red light significantly affected fresh matter content in 15 of them compared to a spectrum with a higher proportion of blue light [22].

The spectral composition of light also has a significant effect on the nutritional value of Brassicaceae microgreens [23]. In some research, a high level of blue light in the spectrum increased the total anthocyanin content of most microgreen species [24]. In a study by Samuolienė et al. [25], increasing the proportion of blue light from 0% to 33% increased the total carotenoid concentration in beet (red leaf) microgreens but not in green leaf microgreens (parsley or mustard). In another study, five different species belonging to the genus *Brassica* were studied but only pak choi showed a significant effect of light quality on total carotenoids and chlorophyll content. This study showed that the *Brassica* species responds to the properties of light and the response can vary between species and cultivars [26]. Due to the high variability in the response of different plant species, even from the same genus, to the applied light spectrum, in our study, we wanted to focus on one species but with different leaf pigmentation.

The present study aims to determine the effect of the spectral composition of varying proportions of red and blue light on the morphological characteristics, fluorescence parameters, and nutritional value of the microgreens of two kale cultivars with green (‘Kapral’) and red (‘Scarlet’) leaves.

## 2. Results and Discussion

### 2.1. Morphological Parameters

Dry mass (DM) depended on both light type and kale cultivar (Table 1). An increased proportion of blue light as well as red light had a positive effect on DM. For these combinations, the DM was from 40 to more than 50% higher compared to W light. At the same time, greater dry mass was stimulated by blue light in green kale leaves and by red light in red kale leaves. The lowest DM value was obtained under W light for both cultivars. The highest DM value was obtained for the green-leaved cultivar under W + B light [27].

The highest Chlorophyll Content Index (CCI) was obtained for the red-leaved cultivar grown under W + R light. Interestingly, this cultivar grown under W light also had the lowest CCI value. Blue light (W + B light) also resulted in a low CCI value for both cultivars.

Chlorophyll biosynthesis is regulated by blue light photoreceptors (i.e., cryptochromes) as well as phytochrome proteins that interact with red and far-red (FR) light [28,29]. Furthermore, red light at 660 nm promotes the activation of phytochrome B and, consequently, the involvement of this protein in chlorophyll biosynthesis [30]. In this study, red-leaved kale showed greater sensitivity to the increased red light contribution to the spectrum, exhibiting the highest CCI value compared to the other combinations. In contrast, cultivation in the spectrum with increased blue light negatively affected the CCI value regardless of the kale leaf colour except for red kale under W light. This could be related to the low percentage of red light in this spectrum. White light did not positively affect the CCI value in red kale in contrast to green kale either, where the cultivar obtained the highest CCI content under this spectrum. The results show that red light might significantly stimulate chlorophyll formation but in red kale cultivars. In other studies on red lettuce, chlorophyll content increased with increased blue light, which was not observed for green lettuce [31]. In contrast, in a study by Ying et al. [24], increasing blue light (from 5 to 30%) had no effect on the chlorophyll content of kale (*Brassica napus* “Red Russian”) microgreens, which could also be due to decreasing red light. According to these authors, reducing the proportion of red light at the expense of enhancing blue light may have reduced the activation of phytochrome-mediated regulation of chlorophyll biosynthesis in Brassicaceae microgreens. The cited examples and the authors’ own research show that the effect of blue and red light on chlorophyll levels in plants at an early stage of development seems to be species- and cultivar-dependent, especially on the pigments contained in the leaves. It is worth noting that higher chlorophyll content for red kale under W + R light was associated with a higher DM yield and greater leaf area. Such correlations are not apparent for green kale.

Leaf length and width were highly dependent on the cultivar. The ‘Kapral’ cultivar with green leaves had a significantly lower value for these parameters compared to the ‘Scarlet’ cultivar. Only cultivation under W + R light resulted in both cultivars having similar leaf lengths. In general, the addition of red light resulted in a significant shortening of the leaf length, especially of the petiole in both cultivars. The lowest length and width were obtained for green kale when grown under W + B light.

Many researchers have shown that a high proportion of blue light in the spectrum strongly inhibited hypocotyl elongation [27,32]. However, in the present study, leaves with significantly shorter petioles in both cultivars were obtained for the combination with the highest proportion of red light. At any rate, the cultivar with green leaves had the shortest leaf length under W + B light. The red cultivar was not sensitive to the growth-inhibiting effect of blue light. Also, in other studies, the leaf length of red basil was significantly shorter under R light compared to white and blue light [33]. Interestingly, in a study by Brazaityte [34], the greatest kale hypocotyl length was obtained under both red and blue monochromatic light. In contrast, in a study on green and red lettuce, the greatest leaf length for both cultivars was obtained under W light compared to a spectrum with an increased proportion of blue or red light [35].

Stem elongation is an important element in the production of microgreens, facilitating plant harvesting [36]. At least two photoreceptor systems—phytochromes and cryptochromes—are involved in mediating the increase in elongation through light [37]. Recent studies indicate that, in addition to low-activity phytochrome, inactivated cryptochrome and activated phototropin may also contribute to this process [38]. Phytochromes are red and far-red light receptors (activated by red light and deactivated by far-red light) and cryptochromes and phototropin are among the blue light receptors. Consequently, both red and blue light can mediate stem elongation through relevant receptors [39]. The small leaf length under W + R light could also be due to the low amount of FR relative to R light in this combination. A higher proportion of FR in the spectrum is known to increase plant length and biomass production in, for example, red lettuce [40,41].

Microgreens with large leaf sizes tend to be more attractive to most consumers, so choosing the right light to stimulate leaf and cotyledon area growth is very important. Boosting the white spectrum with both red and blue light resulted in a larger leaf area. At the same time, the best result was obtained for red kale under W + R light. In general, for the ‘Scarlet’ (red) cultivar, a significantly larger leaf area was obtained compared to the ‘Kapral’ (green) cultivar except when grown under W + B light. In a study by Brazaityte [34], the spectrum with different proportions of blue and red light had no effect on the leaf area of kale microgreens.

The specific leaf area (SLA) values obtained varied widely and depended primarily on the spectrum used. SLA shows a plastic response to light exposure through changes in leaf thickness. The thickest leaves were obtained under W + B light but also under W light. Higher proportions of blue light are associated with the development of “sun leaves” and therefore with thicker leave blades and palisades [42]. According to these authors, such a response is to increase the surface area available for light collection and the penetration of light to the chloroplast level probably involving cryptochrome as well as phototropin, i.e., blue light photoreceptors. Under W + R light, the plants had thin leaves and high SLA as this is how the plants optimise light interception.

The results show that blue light promoted leaf development and dry matter accumulation for green kale only. Such effects could be attributed to better CO_2_ fixation by this cultivar as blue light controls the opening of the stomata [15]. Green leaves were also likely to be more responsive to blue light, which could result in higher nitrogen content in leaves exposed to blue light following an increase in nitrate reductase activity [43].

The quality of light significantly influenced leaf development. The light spectrum used modified the leaf shape index to a greater extent than the cultivar. The highest values were obtained under W light. Enhancement of the spectrum with red light resulted in the smallest value of this index: about 40% lower compared to W light. This could be related to the fact that under white light, both cultivars formed longer leaves (including petioles) and smaller leaf areas showing typical shade avoidance responses [44]. On the other hand, the low value of this index under W + R light may have been due to the high R/FR ratio in this combination compared to other combinations. According to Lee et al. [41], the proportion of far-red light in the spectrum and the low R/FR ratio results in an increased leaf shape index. In the study by Son and Oh [31], the leaf shape index was significantly higher in the spectrum without B light compared to other treatments containing B light. In the current study, the higher value of the leaf shape index was under W light and W + B light indicating that leaf expansion occurred through an increase in leaf length rather than leaf width. According to Gould et al. [45], the photosynthetic properties of red leaves are comparable to those of shade-adapted plants. In the current study, the characteristics of shade-adapted plants, such as large and thin leaves with high chlorophyll content could be observed for red kale only under W + R light.

### 2.2. Fluorescence Parameters

**Figure 1:**
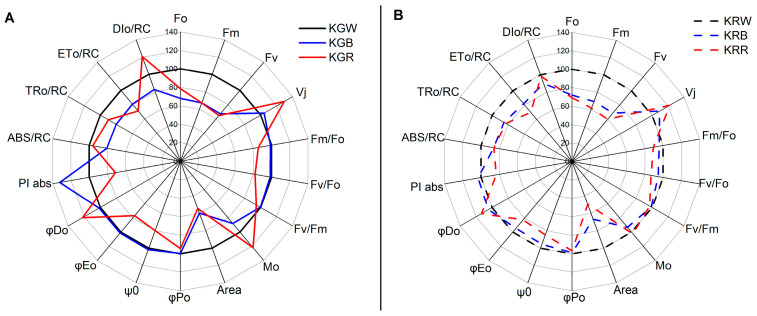
Spider plot of selected fluorescence parameters characterising the changes in PSII of green (**A**) and red (**B**) kale microgreens cultivated under different spectral compositions of light. All values are shown as per cent of the control (W light) (W light = 100). The parameters are divided into the following groups: energy fluxes, quantum yields and efficiencies, specific energy fluxes (calculated per single RC), and generalised parameters of PSII functional activity, i.e., performance indexes. (KGW—green kale under white light, KGB—green kale under white + blue light, KGR—green kale under white + red light, the same for KR—red kale). The presented data are mean values based on 20 replicates.

The light energy absorbed by chlorophyll *a* is largely used to synthesise ATP orNADPH (non-cyclic electron transport) during the light phase of photosynthesis (cyclic electron transport). The excess energy is converted into heat, with some of it being re-emitted as light [46]. Fluorescence is released by healthy leaves and is almost entirely made up of chlorophyll particles which are located mostly in photosystem II (PSII). As a result, it can be used as a predictor of its functionality or the plant’s health and vitality [47]. For the identification of stress states in plants, fluorescence methods are used to monitor physiological processes in plants, particularly photosynthesis [48]. The intensity of photosynthesis is inversely related to the amount of fluorescence. For many years, chlorophyll fluorescence has been employed to evaluate plant photosynthetic performance in a non-invasive manner [49]. Since the PSII structure is particularly vulnerable to stress, fluorimetric methods can be utilised to track variations in PSII reactions [50]. The functioning of the photosynthetic apparatus can be assessed based on chlorophyll *a* fluorescence parameters, with a particular focus on various processes related to PSII photochemistry and whether the plant has been under stress conditions [48,51]. Inappropriate light parameters can be stress factors for plants causing photoinhibition and/or damage to the photosynthetic apparatus. One such parameter is the spectral composition of light under artificial lighting conditions. In the present study, it was shown that, depending on the kale cultivar, the spectral composition of light has a different effect on PSII photochemical efficiency parameters (Figure 1A,B). The analysis of PSII function, assessed through PSII photochemical efficiency parameters, showed that the spectral composition of W + R light can lead to chloroplast dysfunction in the green kale cultivar.

Too much red light caused a deterioration in the PSII vitality index as indicated by a decrease in PI abs (performance index for energy conservation from excitation to the reduction in intersystem electron acceptors) parameter values. Previous studies have shown that differences in PI abs values can be attributed to genetic differences, physiological traits, and environmental conditions [52,53]. In addition, more closed (Vj) and faster closing (Mo) reaction centres were found under W + R light, indicating a disruption in PSII function [37]. This fact is also confirmed by the impaired efficiency of the water-splitting reaction, including oxygen release, on the donor side of PSII (lower value of the Fv/Fo parameter) [54]. The increase in the PI abs in response to abiotic (light) stress under W + B light treatment was mostly due to an increase in primary photochemistry efficiency and photosynthetic electron transport photochemical efficiency, both of which were related to a lower DI0/RC (total energy dissipated per reaction centre (RC) as heat, fluorescence, and energy transfer to PSI) [55]. One of the protective mechanisms of the photosynthetic apparatus, and particularly PSII, against stress-induced damage is the slowing down of electron transport from the reaction centres to the plastoquinones [49,56]. The lowest electron transport rates (ET0/RC) with a concomitant increase in energy dissipation at the cost of heat (DIo/RC) were found in W + R light in both cultivars (Figure 1A,B). Chlorophyll minimum fluorescence (Fo), variable fluorescence (Fv), and chlorophyll maximum fluorescence (Fm) values decreased with the increasing amount of blue or red light in the spectrum. Fo are the minimum fluorescence levels when all the antenna pigment complexes associated with the photosystem are assumed to be open (dark-adapted) [45]. An increase in Fo represents any difficulty and degradation in photosystem II (D1 protein and another part of the PS) or any disruption of energy transfer to the reaction centre [57]. In the present study, there was no effect of the spectral composition of the light on the Fv/Fm parameter. This suggests that kale plants were not photosynthetically stressed under W + R or W + B light treatments. However, the absence of differences between cultivars and the spectrum used confirms the low sensitivity of this parameter to changes in the photochemical performance of PSII [58].

**Figure 2:**
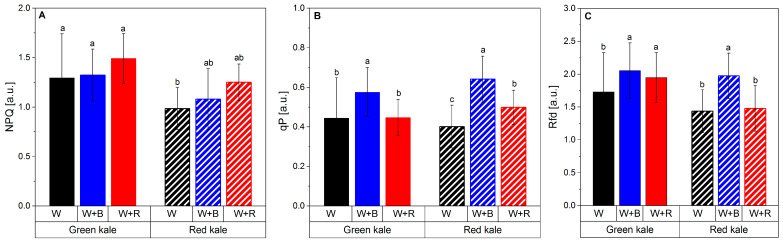
Changes in the non-photochemical fluorescence quenching (NPQ): (**A**) photochemical quenching in the relative reduction state of QA reflecting the fraction of open PSII reaction centres (qP) (**B**) and vitality index of PSII indicative of interactions between the light-stage reactions activated by PAR absorption and the dark reactions of photosynthesis (Rfd) (**C**) in green and red kale microgreens cultivated under different spectral compositions of light. Columns with different letters are statistically different for different lights and types of kale (*p* < 0.05). Mean values from two cycles represent the average of six plants per replication (experimental unit) and four replications per treatment in each cycle.

The negative effect of excessive red light on the efficiency of the photosynthetic apparatus is also evidenced by the higher value of the NPQ parameter (nonphotochemical quenching per reaction centre of PSII) and the low value of qP (photochemical quenching) (Figure 2A,B).

In the case of red kale, there was no such strong effect of the spectral composition of the light on the efficiency of the photosynthetic apparatus. The variations observed in NPQ values can be attributed to genetic and physiological differences between the cultivars. Genetic variations can influence the expression and activity of proteins involved in non-photochemical quenching mechanisms, affecting the overall quenching capacity of the photosynthetic apparatus. Physiological factors, such as differences in pigment composition and abundance as well as the efficiency of energy dissipation mechanisms can also contribute to the variation in NPQ values between cultivars [59]. In summary, adding too much red to the LED light spectrum (W + R) impairs photosynthetic efficiency and a variant with more blue light (W + B) seems preferable, especially for green kale. In this cultivar, too much red light caused a deterioration not only in fluorescence parameters but also in morphological parameters like DM yield or leaf area. No such correlations were found for the red cultivar. This may have been attributed to the high relative chlorophyll content of red kale compared to green kale under W + R light.

### 2.3. Chemical Composition

Fourier transform Raman (FT-Raman) spectroscopy is an effective tool for analysing the chemical composition of biological material [60,61,62,63]. The technique provides a fast, inexpensive, and non-invasive method for obtaining chemical characterisation of a biological sample. Biochemical compounds, i.e., mono- and oligosaccharides, fatty acids, amino acids, proteins, flavonoids, and carotenoids, can be identified from the vibrations of chemical bonds characteristic of individual functional groups [64,65]. FT-Raman analysis makes it possible to assess changes in the proportions of the main organic compounds not only between species but also in relation to environmental conditions.

**Figure 3:**
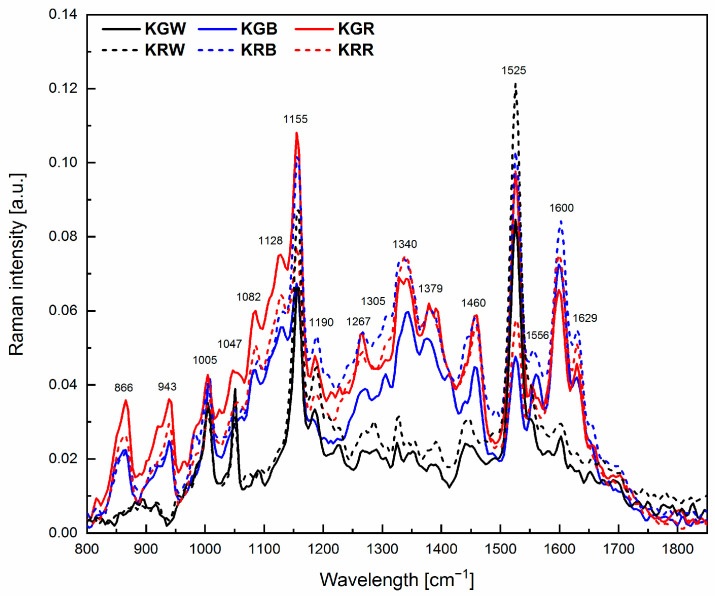
Raman spectra for microgreens grown in different spectral compositions of light together with the marked positions of the peaks characteristic of the relevant functional groups (carotenoids, polysaccharides, chlorophyll, lipids, carbohydrates, flavonoids, and proteins). (KGW—green kale under white light, KGB—green kale under white + blue light, KGR—green kale under white + red light, the same for KR—red kale). The spectra are means from 5 independent measurements.

Changes in the chemical composition of microgreens can be determined by comparing Raman spectra which show specific marker bands characteristic of individual compounds (Figure 3, Table 2). The differences in intensities of the individual peaks observed in the spectrum are indicative of the different content of a particular compound.

The Raman spectra presented for the investigated microgreens had a clearly visible so-called carotenoid triplet, i.e., three bands at 1525, 1155, and 1005 cm^−1^. Another characteristic cluster was the bands at 1556, 1379, 1267, and 1047 cm^−1^ originating from chlorophyll. Phenolic bands were also found at 1600 and 971 cm^−1^ as well as lipid bands at 1460, 1305, and 1267 cm^−1^. Polysaccharide bands were found at 866–943 and 1082 cm^−1^. An analysis of the spectra showed a poorer chemical composition of both microgreens growing under white light compared to the other light combinations. In the Raman spectra of microgreens grown under blue or red light spectra, distinct peaks originating from polysaccharides, carbohydrates (866, 943 and 1340 cm^−1^), and flavonoids (1600 cm^−1^) and additional bands originating from carotenoids (1128 cm^−1^), as well as proteins (1629 cm^−1^), were identified.

**Figure 4:**
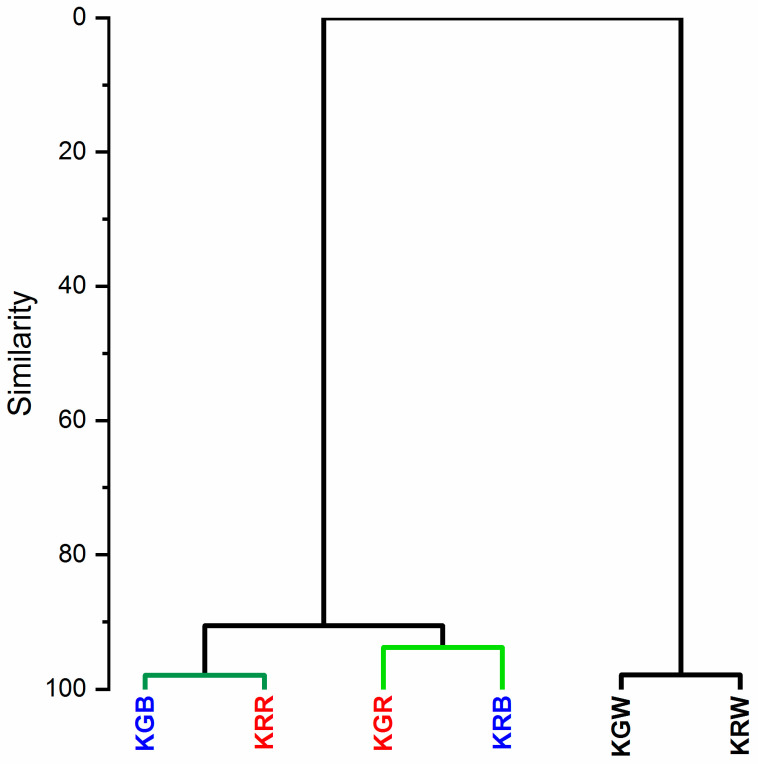
The hierarchical clustering shows the relation to the spectral composition of light. (KGW—green kale under white light, KGB—green kale under white + blue light, KGR—green kale under white + red light, the same for KR—red kale). HCA was performed based on the whole FT-Raman spectra range of 800–1850 cm^−1^ which gave more than 270 variables.

The hierarchical clustering analysis (HCA) showed two main clusters grouping for differences in the chemical composition of the leaf (Figure 4). Plants growing under white light (KGW and KRW) belong to one cluster with a similar chemical composition of leaves (98%). The second cluster was microgreens growing under modified light. Within this cluster, the similarity of the chemical composition of the leaves was at the level of 90%. Interestingly, this group was split into two smaller clusters. One cluster with a similar chemical composition at the level of 92% comprised the leaves of the KGR and KRB and the other one had a chemical composition 98% similar to the KGB and KRR. This analysis showed that in the case of modified light, the spectral composition had a different effect on the tested cultivars of microgreens (green and red). The light for growing microgreens should be selected according to the colour of their leaves. This is related to the different composition of pigments (carotenoids and chlorophyll) in the leaves. Microgreens with green leaves that grew under W + R light had a similar composition of chlorophyll and carotenoid pigments to kale with red leaves under the W + B light (Figure 4) This is confirmed by the more intense FT-Raman bands coming from these pigments for the above-described leaf colour and light colour relationships (Figure 3). The greater effect of blue light on red kale may be related to the fact that blue light is specifically used as the wavelength required for anthocyanin synthesis, for example in red lettuce [71]. The expression of genes that induce anthocyanin synthesis is induced by blue light and is known to be mediated by cryptochrome (Cry1) [72]. In contrast, light that can interfere with anthocyanin expression includes green, red, and far-red light [73].

Numerous studies have shown that red and blue LED light can affect the accumulation of bioactive compounds in microgreens [74,75]. Even though the results obtained by authors vary widely and depend on many factors, the most important factors are the plant species and growing conditions [25,76]. Studies on various species have shown that blue light has a major effect on carotenoid synthesis [71,77]. Its effect can be linked to an increase in the transcription of carotenoid genes, for example, in *Brassica* sprouts grown under a spectrum with a high proportion of blue light [26]. However, only in the case of pak choi did this result in higher carotenoid content. According to some researchers, an increased percentage of blue light resulted in a reduced carotenoid reflectance index (CRI2) value in kale microgreens [34], produced a greater accumulation of carotenoids in kale sprouts [78], or had no effect on carotenoid content in kale microgreens [24]. The effect of blue light depends also on light conditions. Blue light alone is not enough to increase carotenoid levels and other wavelengths of the visible light spectrum besides blue light are necessary for carotenoid enrichment for example in pak choi and other *Brassica* species [77]. In other research, the total and individual carotenoids increased by 20–44 and 10–55%, respectively, in dose-dependent increasing amber-blue and decreasing red light in most Brassica microgreens [79]. Also, green (520 nm), yellow (595), and orange (622 nm) supplemental light spectra to standard illumination had positive impacts on the total carotenoid content in mustard; however, contents decreased in red pak choi [80]. The effect of red LED light on carotenoid accumulation may also depend both on the species and the light conditions during cultivation [81]. The lutein content of fresh kale leaves is significantly affected by red light (660 nm) and the β-carotene content by blue light (440 nm) [82]. Since young kale leaves primarily contain a large amount of lutein [83], the significant effect of red light on the carotenoid content of kale microgreens may be due to this.

Based on the results, it can be concluded that in the case of red kale, red light had a greater effect on growth and blue light on the content of active compounds. The opposite situation was in green kale. Blue light affected morphological characteristics to a greater extent and red light affected the content of active compounds. Red light to a greater extent than blue light caused the deterioration in fluorescence parameters in both varieties. However, the response of blue kale was stronger and the reduction in the vitality of the PSII index affected the deterioration in morphological characteristics.

In microgreen cultivation, leaf area is an important morphological parameter. Looking at this aspect, it is worth using additional red LEDs in the cultivation of red kale and blue LEDs in the cultivation of green kale. On the other hand, wishing to increase the nutritional value of the plants, one could enhance the white light with additional blue LEDs for red kale and red LEDs for green kale. However, these diodes could be applied to the last few days of the cultivation; this would require further research.

## 3. Materials and Methods

### 3.1. Plant Material and Growth Conditions

In the current experiment, two kale cultivars (*Brassica oleracea* var. *acephala* L.) were used for the production of microgreens, ‘Kapral’, a green-leaved cultivar, and ‘Scarlet’, a red-leaved one. Seeds were obtained from a commercial source (“W. Legutko” company, Jutrosin, Poland). The kale cultivars were grown in special plastic tray vessels (30 × 50 × 5 cm) filled with peat substrate (sphagnum peat) for the production of the seedlings (Klasmann-Deilman, Geeste, Germany). The average amounts of nutrients (mg L^−1^) in the substrate entails a neutralising minerals and fertiliser PG Mix N:P_2_O_5_:K_2_O at a proportion of 14:16:18 and Mg at a density of 1.0–1.3 kg/m^3^; the pH of the substrates ranges from 5.5 to 6.5 ± 0.2 (in H_2_O).

The broadcasting method of sowing was used, with 3 g of seeds sown per tray (four trays for each treatment and one tray corresponding to one replicate). Subsequently, a lid was placed on top of the containers and the trays were left in darkness until germination (3 days). The average germination percentage was 98%.

The experiment was performed in a controlled-environment growth chamber. The temperature in the growth chamber was maintained at 25 °C until the seeds emerged. During the growing period, the day/night temperature was 21/17 ± 2 °C. The relative air humidity was maintained at 50–60%. The photoperiod was 16/8 h (day/night). Plants were irrigated on average every two days, as needed.

### 3.2. Treatment with Light

**Figure 5:**
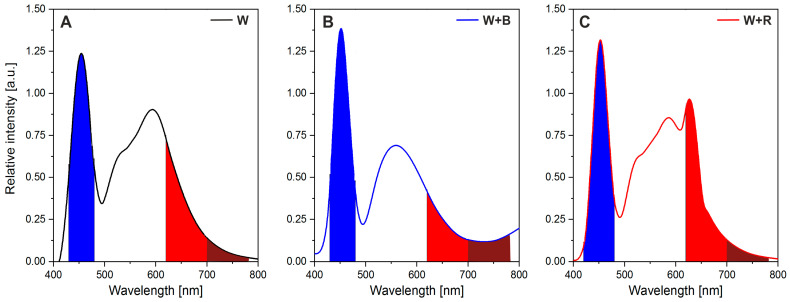
The spectral composition of light-emitting diodes (LED) used during microgreens growth with areas of blue (430–480 nm), red (620–700 nm), and far-red (700–780 nm) light highlighted. (**A**) One hundred per cent of white light, (**B**) white light enhanced in the blue light range, and (**C**) white light enhanced in the red light range.

The three light treatments were used in the current experiment: 100% white light (W), white light with red light (W + R), and white light supplemented with blue light (W + B) (Figure 5). The photosynthetic photon flux density (PPFD) from the top of the plants for white light in the W + R and W + B light combinations amounted to about 170 µmol m^−2^ s^−1^ (±14 SD) and, additionally, 60 µmol m^−2^ s^−1^ (±8 SD) for blue (B) and red (R) light, respectively. All LED treatments had an intensity of 230 µmol m^−2^ s^−1^ and the daily light integral (DLI) was about 13.2 mol m^−2^ d^−1^. The specific wavelength range of the visible light was measured for the red (620–700 nm), blue (430–480 nm), and far-red (700–780 nm) according to Malacara [84] (Figure 5). The R/B ratios and R/FR (red/far-red) ratio (in parentheses) for W, W + R, and W + B lights were 0.63 (5.8), 0.75 (7.4), and 0.38 (1.8), respectively. The measurements were made 15 cm under the lamps more or less at the height of the plants’ tops. The LED panel was arranged in the growth chamber so as to ensure a homogeneous light treatment. The PPFD was measured with a PAR-10 quantum sensor (Sonopan, Białystok, Poland). The spectral distribution of light treatments was measured with a BLACK-Comet CXR UV-VIS spectroradiometer (280–900 nm, StellarNet Inc., Tampa, FL, USA).

### 3.3. Measurement and Collection of Growth Parameter Data

The harvest was gathered when cotyledons and the first true leaves were produced by the plants. The time for the harvest was fourteen days after germination and the plants were harvested with scissors to cut at the lowest part of the stem.

The length (including petiole) and width of leaves were measured using a measuring ruler in centimetres. These parameters were used to calculate the values of the leaf shape index, i.e., the leaf length-to-width ratio. The fresh weight and dry weight were checked with an electronic weighing balance (g, parameters were measured using laboratory scales WTB200, R: 0.001 g; Radwag Poland). The leaf area was first measured using a scanner and then the Skwer program was used to calculate the leaf area. Dry mass (DM, mg) was measured by weighing dried microgreens. Plants were weighed after weighing for 24 h at 100 °C [85]. The leaf rosette area and dry mass [m^2^ kg^−1^ DM] were used to calculate specific leaf area (SLA). All measurements were taken on ten fully developed plants randomly selected from each replication.

The chlorophyll content index (CCI) was measured with the use of the OSI CCM-200 plus Leaf Chlorophyll Meter (ADC BioScientific Ltd., Hoddesdon, UK). Chlorophyll fluorescence was measured with the use of the OS1-FL modulated fluorometer (Opti-Sciences, Hudson, NH, USA) half an hour after the termination of the period of exposure to light. The measurements were made on a fully grown leaf for six plants from each replicate.

The following parameters were selected for the analysis [86]:

- Basic parameters of chlorophyll fluorescence:

Fo—minimal fluorescence, when all PSII reaction centres (RCs) are open; Fm—maximal fluorescence, when all PSII RCs are closed; Fv—maximal variable fluorescence, which is measured after dark adaptation. The extent of Fv is related to the maximum quantum yield of PSII.; Vj—information about the number of closed RCs in relation to the total number of RCs that can be closed; Fm/Fo—the ratio of maximum fluorescence to zero-time fluorescence. The low ratio Fm/Fo could indicate the destruction of PSII; Fv/Fo—ratio of rate constants for photochemical reaction and nonphotochemical deactivation of PSII excitations; Fv/Fm—maximum photochemical quantum PSII after dark adaptation; Mo—approximated initial slope (in ms^−1^) of the fluorescence transient normalised on the maximal variable fluorescence FV; Area—the area above the fluorescence induction curve, is proportional to the pool size of electron acceptors in PSII.

- Quantum yields or flux ratios:

φPo—maximum quantum yield of primary photochemical reactions that indicates the probability of trapping the energy of absorbed photons (or excitons migrating over the antenna) by PSII reaction centres. In plants stressed by heat or high-intensity light, the φPo values are usually lowered; ψ0—the probability of electron transport beyond Q_A_^−^, i.e., the efficiency with which the exciton trapped by an RC drives the electron along ETC beyond Q_A_^−^;

φEo—quantum efficiency of electron transfer from Q_A_^−^ to the electron transport chain beyond Q_A_^−^; φDo—quantum efficiency of energy dissipation.

- Performance indices:

PI abs—performance index (potential) for energy conservation from excitation to the reduction in intersystem electron acceptors.

- Specific energy fluxes per Q_A_-reducing PSII reaction centre:

ABS/RC—the light energy absorbed by the PSII antenna photon flux per active reaction centre; TRo/RC—total energy used to reduce Q_A_ by the unit reaction centre of PSII per energy captured/trapped by a single active RC; ETo/RC—the rate of electron transport through a single RC; DIo/RC—total energy dissipated per reaction centre (RC) as heat, fluorescence, and energy transfer to PSI.

- Parameters of nonphotochemical and photochemical fluorescence quenching:

NPQ—nonphotochemical quenching per reaction centre of PSII. This parameter is associated with heat losses. For the majority of healthy plants, NPQ values are 0.5–3.5 although they differ in various species or plants cultivated at different growth conditions; qP—photochemical quenching, this is the fraction of light energy consumed by the open centres for photosynthetic reactions with respect to the total amount of energy absorbed by PSII; Rfd—vitality index of PSII. This parameter is indicative of interactions between the light-stage reactions activated by PAR absorption and the dark reactions of photosynthesis. This parameter is diminished when the balance between photochemical reactions in thylakoids and the rates of enzymatic reactions in the chloroplast stroma is disturbed.

- FT-Raman Spectroscopy Measurements

An analysis of the chemical composition of microgreens growing under different spectral compositions of light was performed using a Nicolet NXR 9650 FT-Raman spectrometer equipped with a laser emitting a 1064 nm beam. Microgreens were freeze-dried prior to measurement. The freeze-dried leaves were placed on the spectrometer table so that the laser beam fell on a leaf centre during the measurement. Measurements were carried out at an aperture of 50 and a spectral resolution of 8 cm^−1^. The spectra were recorded at a laser power of 1.0 in the range from 800 to 2000 cm^−1^. A total of 64 scans were collected for the spectrum. Measurements were performed in 6 repetitions for each light composition and object. All the spectra obtained were analysed with Omnic 8/Thermo Scientific software (Thermo Scientific, Walthman, MA, USA) and OriginLab 2020 software (OriginLab Corporation, Northampton, MA, USA) using baseline correction, smoothing (7 points), and normalisation with vector normalisation functions. In addition, hierarchical clustering analysis (HCA) was performed for the entire range of the Raman spectrum to compare the chemical composition of the leaf of the analysed species of microgreens. The Euclidean distance was used in the HCA. The distance between similar groups was measured by means of the Ward algorithm. OriginPro 2020 software was used for the HCA analysis.

### 3.4. Experiment Design and Statistical Analysis

The research was conducted as a two-factorial experiment in four replicates as an independent design. Photosynthetic parameters were analysed separately for each cultivar. One tray was treated as one replicate. The investigations were conducted in two cycles (replications one after another). The results were the means of the two cycles. The data were analysed with ANOVA. Differences between the means were estimated with the Newman–Keuls test at a significance level α = 0.05. The data were analysed statistically with the Statistica program (StatSoft, Kraków, Poland).

## 4. Conclusions

The study showed that the applied spectrum had different effects on the various parameters studied in red and green kale. The red light had a greater effect on the morphological characteristics of the red cultivar, especially DM yield, leaf area and SLA. In the case of this cultivar, a deterioration in the PSII vitality index under red light did not affect the morphological traits. The content of active compounds was more affected by blue light. 

Blue light influenced more on the morphological traits of green kale. On the other hand, a higher content of active compounds in the Raman spectra analysis in this cultivar was obtained with an increased amount of red light in the spectrum. However, the higher amount of red light in the spectrum caused a decrease in the performance of fluorescence parameters in blue kale which significantly affected morphological traits.

Our results demonstrated that red and blue light could be strategically used to enhance the nutritional value and growth of kale microgreens grown under white light. The study revealed a different sensitivity of kale with red versus green leaves to the spectrum used. Therefore, further research is needed. Firstly, concerning the possibility of using more red/blue light at different stages of microgreen growth depending on the cultivar. For red-leafed kale cultivars, a spectrum with increased red light can be used at the initial stage to shape morphological traits and at the next stage with an increased blue light spectrum to increase the nutritional value of the plants. In green-leafed cultivars, the order of application of each light colour would be reversed. The results also show that for obtaining high-quality kale microgreens, an appropriate proportion of red and blue light in white light is very important. Therefore, secondly, it would be necessary to conduct studies with more combinations of R/B light ratios in the spectrum in order to find a balance that, for cultivars with different leaf colours, would provide both optimal growth and performance of fluorescence parameters and a high nutritional value.

## Figures and Tables

**Table 1:** Morphological parameters of green and red kale microgreens.

Biometric Parameters	Light Treatments						
	White (W)		White + Red (W + R)		White + Blue (W + B)		Mean for Kale
	Green Kale	Red Kale	Green Kale	Red Kale	Green Kale	Red Kale	
DM * (per plant, mg)	2.19 d **	2.15 d	3.61 bc	4.17 ab	4.63 a	3.12 c	G *: 3.48 a
Mean for light	2.17 b		3.89 a		3.87 a		R: 3.15 a
CCI	14.3 b	10.1 d	13.3 c	18.1 a	12.2 d	12.1 d	G: 13.3 a
Mean for light	12.2 b		15.6 a		12.1 b		R: 13.4 a
The leaf length (cm)	3.1 c	3.7 a	2.2 d	2.1 d	1.4 e	3.4 b	G: 2.2 b
Mean for light	3.4 a		2.2 c		2.4 b		R: 3.1 a
The leaf width (cm)	2.4 c	2.8 b	2.7 b	2.8 b	1.5 d	3.6 a	G: 2.3 b
Mean for light	2.6 a		2.8 a		2.6 a		R: 3.1 a
Leaf area (cm^2^)	9.40 e	11.07 d	12.10 cd	14.62 a	13.25 b	12.94 bc	G: 11.58 b
Mean for light	10.23 b		13.36 a		13.10 a		R: 12.88 a
SLA * (m^2^ kg^−1^)	21.68 c	15.32 c	39.40 a	30.61 b	17.59 c	17.51 c	G: 26.22 a
Mean for light	18.51 b		35.01 a		17.55 b		R: 21.15 a
Leaf shape index	1.28 a	1.35 a	0.81 c	0.79 c	0.95 b	0.96 b	G: 1.01 a
Mean for light	1.31 a		0.80 c		0.95 b		R: 1.03 a

* DM—Dry Mass, CCI—Chlorophyll Content Index, SLA—Specific Leaf Area, G—green kale, R—red kale. ** Data followed by the same letter do not differ significantly at α = 0.05 for each parameter and separately for means. Mean values from two cycles represent the average of ten plants per replication (experimental unit) and four replications per treatment in each cycle.

**Table 2:** Summary of wavelengths in the Raman spectrum at which peaks characteristic of individual compounds occur.

Wavelength [cm^−1^]	Chemical Compounds	References
866	Monosaccharides	[66]
943	Polysaccharides	[66]
971	Phenols	[67]
1005	Carotenoids	[68]
1047	Chlorophyll *a*	[66]
1082	Polysaccharides	[66]
1128	Carotenoids	[68]
1155	β-carotene	[68]
1190	Disaccharides, β-carotene	[68]
1267	Lipids, Chlorophyll *a*	[66]
1305	Lipids	[69]
1340	Carbohydrates	[70]
1379	Chlorophyll *b*	[66]
1460	Lipids	[69]
1525	Carotenoids	[68]
1556	Chlorophyll *a*	[66]
1600	Flavonoids	[66]
1629	Proteins	[66]

## Data Availability

Data are contained within the article.
